# Analysis of the evolution, infectivity and antigenicity of circulating rabies virus strains

**DOI:** 10.1080/22221751.2022.2078742

**Published:** 2022-06-01

**Authors:** Meina Cai, Haizhou Liu, Fei Jiang, Yeqing Sun, Wenbo Wang, Yimeng An, Mengyi Zhang, Xueli Li, Di Liu, Yuhua Li, Yongxin Yu, Weijin Huang, Youchun Wang

**Affiliations:** a Division of HIV/AIDS and Sex-Transmitted Virus Vaccines, Institute for Biological Product Control, National Institutes for Food and Drug Control (NIFDC), Beijing, People’s Republic of China; b Graduate School of Peking Union Medical College, Beijing, People’s Republic of China; c National Virus Resource Center, Wuhan Institute of Virology, Chinese Academy of Sciences, Wuhan, People’s Republic of China; d CAS Key Laboratory of Special Pathogens and Biosafety, Wuhan Institute of Virology, Center for Biosafety Mega-Science, Chinese Academy of Sciences, Wuhan, People’s Republic of China; e Division of Monoclonal Antibody Products, Institute for Biological Product Control, National Institutes for Food and Drug Control (NIFDC), Beijing, People’s Republic of China; f Department of Arboviral Vaccine, National Institutes for Food and Drug Control, (NIFDC), Beijing, People’s Republic of China

**Keywords:** Rabies virus, genetic evolution, antigenic evolution, infectivity

## Abstract

Rabies virus has existed for thousands of years and is circulating in many species. In the present study, a total of 2896 rabies viruses isolated worldwide were phylogenetically classified into ten clusters based on the G gene sequence, and these clusters showed a close relationship with the hosts and regions that they were isolated from. Eighty-three representative G sequences were selected from ten clusters and were used to construct pseudoviruses using the VSV vector. The phylogenetic relationships, infectivity and antigenicity of the representative 83 pseudotyped rabies viruses were comprehensively analyzed. Eighty three pseudoviruses were divided into four antigentic clusters (GAgV), of which GAgV4 showed poor neutralization to all immunized sera. Further analysis showed that almost all strains in the GAgV4 were isolated from wild animals in the America, especially bats and skunks. No significant relationship in terms of phylogeny, infectivity and antigenicity was proved. Amino acid mutations at residues 231and 436 can affect the infectivity, while mutations at residues 113, 164 and 254 may affect the sensitivity to immunized animal sera, especially residue 254. We recommend close monitoring of infectivity and antigenicity, which should be more precise than simple genetic analysis.

Rabies is caused by the rabies virus (RABV), which belongs to the *Lyssavirus* genus of the *Rhabdoviridae* family [1, 2]. The virus has a non-segmented, single-stranded, negative-sense RNA genome with a size of approximately 12 kb, encoding nucleoprotein (N), phosphoprotein (P), matrix protein (M), glycoprotein (G), and the polymerase protein (L) [3–7]. Glycoprotein (G), the only membrane protein, is a type I transmembrane protein [8]. As one of the most important antigens, G protein is responsible for interacting with receptors to enter the host cell and plays a vital role in inducing neutralizing antibodies [9, 10]. Thus, G protein plays an important role in determining the host range and antigenicity [11, 12].

The literature generally recognizes urban, rural, wild, and aerial cycles of rabies maintenance [13–15]. Terrestrial domestic mammal animals (dogs and cats) are responsible for rabies in the urban and rural cycle. Wild carnivores (foxes, wolves and skunks) are responsible for rabies in the wild cycle and bats are responsible for the aerial cycle. Because bats response the aerial cycle, it is implicated in the maintenance and dissemination of RABV in wild, rural, and urban environments [13–15]. Humans can be accidentally infected in any of the above cycles [14, 16].

Previous studies mainly focused on phylogenetic relationships among isolates from four different regions. In Asian countries, RABVs had genetic diversity and its distribution was regional. Among them, the rabies viruses in China had genetic diversity and its distribution was regional, mainly in the southern and southeastern regions, while in India, the rabies viruses mainly belonged to Arctic-like 1a lineage viruses, which was related to geography [17–20]. In American countries, RABV strains had regional differences and varying species specificity. Bats played a pivotal role in viral spread and endemicity, with evolution directed towards adaptation to the host species [13, 21–25]. In Europe, there were local phylogenetic relationships of RABV, with distinct groups associated with particular geographic areas [26, 27]. In African countries [28–30], the evolution of RABVs were host dependent and can be divided into three phylogenetic lineages: Africa 1, 2 and 3. Africa 1 and 2 lineages were isolated from dogs or humans bitten by rabies dogs, while African 3 lineages were related to mongoose species, mainly the Yellow mongoose (*Cynictis penicillata*). The Africa 1 lineage can be divided into two subgroups, Africa 1A and 1B, with Africa 1A found only in North Africa and West Africa, while Africa 1B was found in Southeastern Africa. The Africa 2 lineage was distributed in Central Africa and East Africa. The Africa 3 lineage was isolated from the Republic of South Africa. However, there are very little known about the epidemiology and phylogenetic relationships of RABVs worldwide [28–30].

In previous studies, antigenic characterization was mainly based on monoclonal antibodies against the viral N protein. This method had been used in several countries, and at least 11 antigenic variants (NAgV) had been found so far [31]. The RABVs from different countries or hosts may belong to different antigenic variants. In Mexico, the antigenic type of vampire bats mainly belonged to NAgV11 and NAgV3. The small number of dog isolates and human cases was found to belong to NAgV1. Three skunks were typed as NAgV10 and one with NAgV8. And two bobcat specimens belonged to NAgV7 [32, 33]. In Northeastern Brazil, all the viruses isolated from domestic animals (dogs and cats) belonged to the NAgV2 [31]. In Israel, the RABV virus showed 6 antigen variations, NAgV1-V6, among which AgV1 was the most common antigenic type, and NAgV6 was first identified in 2000.

Although this method based on N protein can differentiate antigenic variants, it cannot represent differences in the antigenicity of G protein, which plays an important role in the prevention and treatment of rabies. We, therefore, used the neutralization assay with vaccine-elicited sera against the RABV strains to map the antigenic evolution of the G protein in this study. This provides a strong theoretical basis for further development and updating of rabies vaccines.

The in vitro infectivity of rabies virus was also an important characteristic that differs among strains. In addition to neurons, RABV can infect a variety of non-neuronal cell types, albeit with lower affinity [34, 35]. Although rabies virus had stronger infectivity to neuronal cells [36], circulating and deposited strains had different infectivity in astrocytes [34]. However, there is almost no research comparing the infectivity of circulating viruses. Therefore, we analyzed the phylogenetic relationships, infectivity and antigenic evolution of circulating rabies virus isolates, and identified key single amino acid mutations which affect their relationship. Our results will facilitate the monitoring of the antigenicity and infectivity of circulating strains and may lay the foundation for further vaccine development and updating.

## Results

### Evolution of circulating rabies virus strains

**Figure 1.**
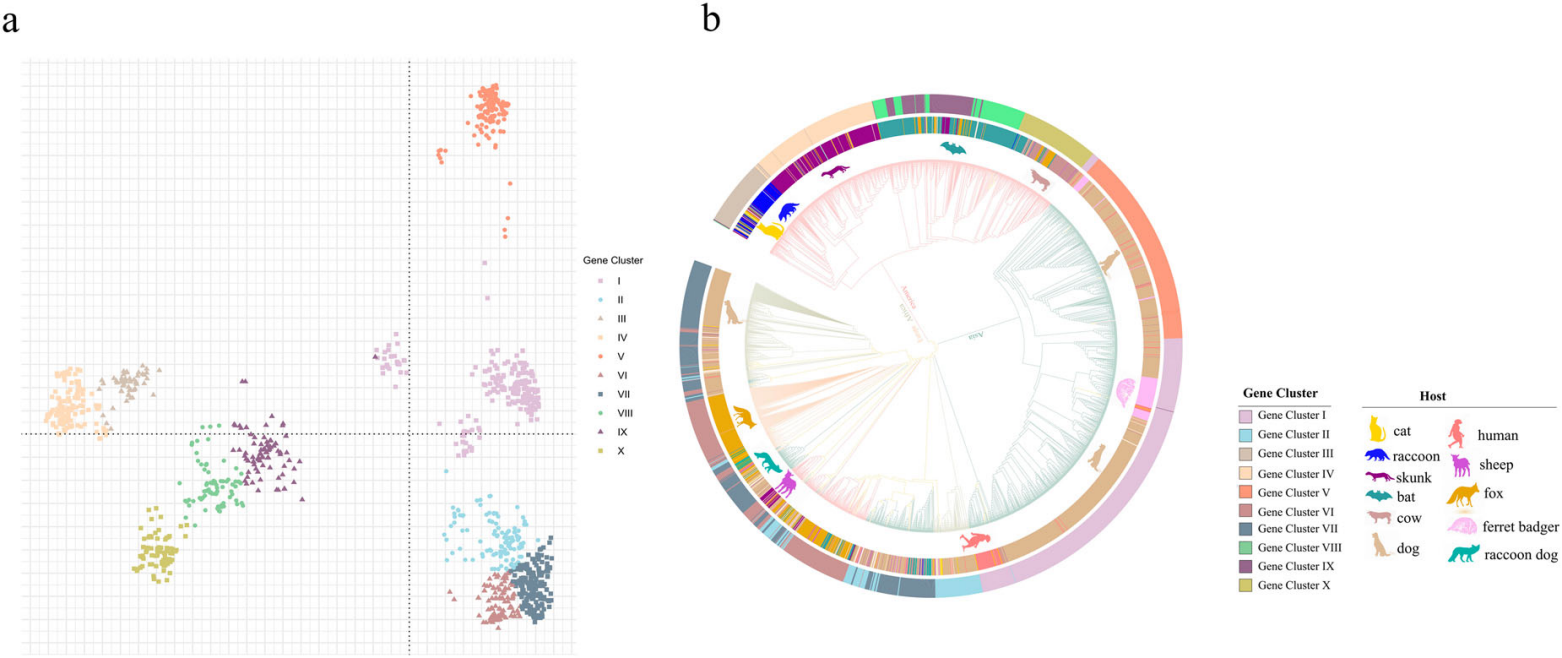
Phylogenetic relationships of circulating rabies virus strains. (a) Phylogenetic tree of 2896 rabies viruses based on G gene sequences. The sequences were classified into ten clusters, named I–X. (b) A phylogenetic tree showing the relationships with the host and region. The branch colours represent the region, and the circles and animal pictures represent the hosts. Canine refers to rabies viruses isolated from dogs; although raccoon dogs and foxes are technically also canines, they are listed separately in this study. Other represents a small number of hosts that are less specific, such as donkey, pig, and some unknown hosts. In the clockwise direction, the circles show RABVs isolated from cats, raccoons, skunks, bats, cows, dogs (canine), ferret badgers, dogs (canine), humans, sheep (ovine), raccoon dogs, foxes, and dogs (canine).

After removal of incomplete, redundant, and ambiguous sequences, a total of 2896 *G* gene sequences of rabies virus were obtained from GenBank, 2890 of which were circulating sequences, eight were reference virus sequences, and two sequences were found among both the circulating and reference viruses. A phylogenetic tree for the 2896 gene sequences was inferred, using Gannoruwa bat lyssavirus (Gene accession number KU244269.2) as the out-group. More information, such as the source countries and hosts, was also presented in this phylogenetic tree (Figure 1 and Figure S1). Thus, this tree showed the phylogenetic relationships, hosts and geographical locations of the viral strains.

To analyze the phylogenetic relationships of the viral strains, we calculated the pairwise distances of all gene sequences, followed by principal co-ordinate analysis (PCoA). The k-means analysis was used to cluster the virus strains according to the PCoA function. The sequences could be classified into ten clusters, named I–X (Figure 1(a)). Figure 1(a) showed the relative phylogenetic distances of the ten gene clusters. Gene clusters II, VI and VII maintain a close mutual evolutionary relationship, as do gene clusters VIII, IX and X, as well as gene clusters III and IV, respectively. By contrast, gene clusters I and V, and especially cluster V, were relatively distant from the other gene clusters (Figure 1(a)).

The gene clusters exhibited geographical specificity, since viruses from the same region tended to cluster together. The strains in gene clusters I and V were almost all isolated from Asia, while the strains in the gene clusters III, IV, VIII, IX, and X were all located in America (Figure 1(b)). By contrast, the strains in gene clusters II, VI, and VII were located across Asia, Europe, Africa and America (Figure 1(b)). In addition to their geographical specificity, strains from the same hosts tended to cluster together. Strains from the raccoon group formed the gene cluster III, while strains from skunks formed the gene cluster IV. The strains in gene clusters VIII and IX were isolated from bats, while gene cluster X mainly contained bovine or bat-related RABVs. Finally, the strains in gene clusters I, V and VII contained RABVs from dogs (Figure 1(b)). Thus, the phylogenetic relationships not only reflected the geographic origins of the strains, but also their host animals.

In addition, the phylogenetic tree also showed that viruses from ferret badgers located in Asia were closely related to viruses isolated from dogs located in Asia, indicating that they may represent early cross-species transmission in Asia (Figure 1(b) and Figure S1). There were a large number of dog-derived strains in Asia, but they only belonged to two gene evolutionary clusters, indicating that Asian dog-derived strains had low genetic diversity (Figure 1(b)). Bat- and skunk-derived rabies virus strains were almost isolated in America (Figure 1(b) and Figure S1).

### Infectivity of circulating rabies viruses

**Figure 2.**
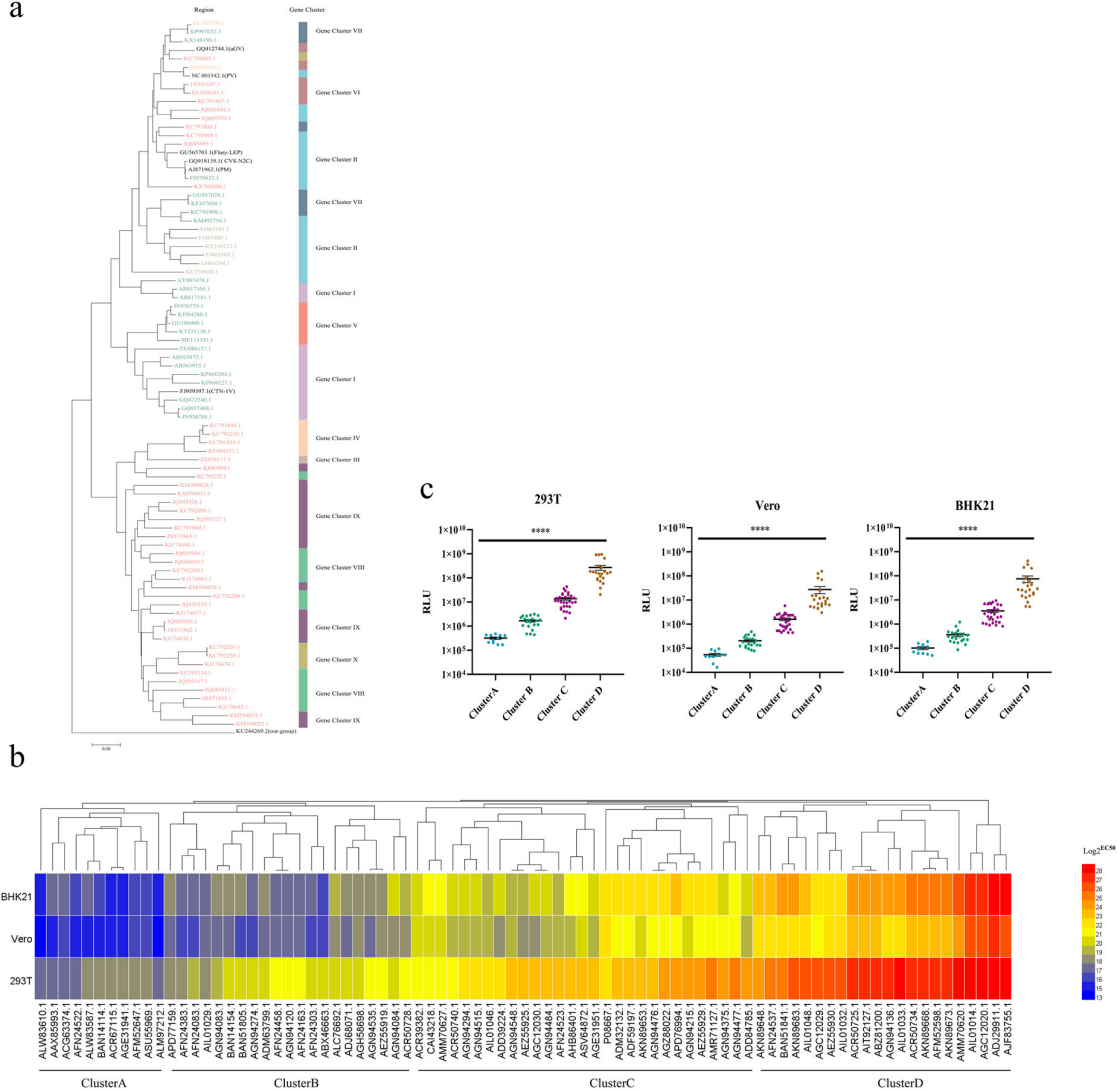
The infectivity of pseudotyped viruses. (a) A phylogenetic tree of 83 representative rabies viruses. (b) Infectivity cluster of 83 representative rabies viruses. (c) The infectivity of the different clusters. The *Y*-axis indicates the RLU values of different strains and represents the mean ± SD. Infectivity of different cluster strains was compared using the rank sum test, differences with *P* < 0.05 were considered statistically significant. *P < 0.05; **P < 0.01; ***P < 0.005; ****P < 0.0001.

Considering host and geographical factors, we selected 77 sequences (Table 1) from the ten gene clusters. In order to analyze whether they represented all the sequences, a phylogenetic tree of 83 sequences (77 circulating sequences and 6 reference strains) was drawn (Figure 2(a)), in which the Gannoruwa bat lyssavirus (gene accession number KU244269.2) was used as the out-group. A comparison of the topological structure with the original phylogenetic trees showed a similar evolutionary structure with the original one, which indicated that the sequences we selected were representative (Figure 2(a)).

**Table 1.** Summary of the characteristics of the 83 rabies pseudoviruses in our library.

Gene accession number	Protein accession number	Host	Country	Gene cluster	Infectivity cluster	Antigenic cluster	Note
GQ412744.1	ADM32132.1	Dog	China	VI	C	1	aGV
FJ959397.1	ACR39382.1	Homo sapiens	China	I	C	3	CTN-1V
GQ918139.1	ADJ29911.1	/	France	II	D	3	CVS-N2C
GU565703.1	ADD84785.1	/	/	II	C	1	Flury-LEP
AJ871962.1	CAI43218.1	/	/	II	C	1	PM
NC_001542.1	P08667.1	Homo sapiens	/	II	C	3	PV
AY987478.1	AAX85993.1	Dog	India	II	A	2	
EU086157.1	ABX46663.1	Homo sapiens	Thailand	I	B	1	
EU293116.1	ABZ81200.1	Tadarida brasiliensis	Argentina	VIII	D	1	
EU886633.1	ACG63374.1	Red fox	Austria	VI	A	2	
FJ465385.1	ACR50725.1	Feline	South Africa	II	D	1	
FJ465388.1	ACR50728.1	Bovine	South Africa	II	B	1	
FJ465394.1	ACR50734.1	Ovine	South Africa	II	D	3	
FJ465400.1	ACR50740.1	Felis nigripes	South Africa	II	C	1	
FJ979833.1	ACR67115.1	White albino mouse	India	II	A	4	
GU186408.1	ADD39224.1	Dog	China	V	C	2	
GU937028.1	ADF59197.1	Racoon dog	South Korea	VII	C	1	
GQ857468.1	ADJ68071.1	Ferret badger	China	I	B	3	
GQ472540.1	ADM63799.1	Canis lupus familiaris	China	I	B	2	
JQ595317.1	AEZ55919.1	Corynorhinus townsendii	USA	VIII	B	2	
JQ595323.1	AEZ55925.1	Myotis yumanensis	USA	VIII	C	2	
JQ595327.1	AEZ55929.1	Myotis austroriparius	USA	IX	C	4	
JQ595328.1	AEZ55930.1	Myotis keenii	USA	IX	D	2	
JN936739.1	AFM52598.1	Dog	China	V	D	1	
JN936788.1	AFM52647.1	Ferret badger	China	I	A	3	
JQ685894.1	AFN24083.1	Striped skunk	USA	II	B	1	
JQ685915.1	AFN24163.1	Lasiurus intermedius	USA	VIII	B	2	
JQ685954.1	AFN24303.1	Spilogale putorius (Spotted skunk)	Mexico	IX	B	1	
JQ685970.1	AFN24383.1	Striped skunk	USA	II	B	4	
JQ685930.1	AFN24458.1	Striped skunk	USA	IX	B	2	
JQ685995.1	AFN24522.1	Gray fox	USA	II	A	1	
JQ685996.1	AFN24523.1	Eptesicus fuscus	USA	VIII	C	2	
JQ686010.1	AFN24537.1	Fox	USA	VIII	D	1	
JX871853.1	AGC12020.1	Homo sapiens	USA	VIII	D	2	
JX871862.1	AGC12029.1	Eptesicus fuscus	USA	IX	D	1	
JX871863.1	AGC12030.1	Parastrellus hesperus	USA	IX	C	2	
JX856107.1	AGE31941.1	Striped skunk	USA	VI	A	1	
JX856117.1	AGE31951.1	Raccoon	USA	III	C	2	
KC595280.1	AGH58698.1	Vulpes vulpes (red fox)	Russia: Lipetsk region	VII	B	3	
KC791807.1	AGN94083.1	Skunk	USA	VI	B	4	
KC791808.1	AGN94084.1	Cow	USA	VII	B	2	
KC791844.1	AGN94120.1	Skunk	USA	IV	B	1	
KC791860.1	AGN94136.1	Parastrellus hesperus	USA	IX	D	1	
KC791939.1	AGN94215.1	Skunk	USA	IV	C	2	
KC791998.1	AGN94274.1	Dog	Afghanistan	VII	B	1	
KC792018.1	AGN94294.1	Pecari tajacu	USA	II	C	1	
KC792099.1	AGN94375.1	Homo sapiens	USA	IX	C	3	
KC792200.1	AGN94476.1	Histiotus montanus	Peru	VIII	C	4	
KC792201.1	AGN94477.1	Homo sapiens	Mexico	X	C	1	
KC792208.1	AGN94484.1	Potos flavus	Peru	VIII	C	4	
KC792239.1	AGN94515.1	Cat	USA	IV	C	3	
KC792259.1	AGN94535.1	Desmodus rotundus	Mexico	X	B	2	
KC792272.1	AGN94548.1	Puma	USA	VIII	C	3	
KF437650.1	AGZ88022.1	Dog	South Korea	VII	C	2	
KF484557.1	AHB86401.1	Skunk	USA	IV	C	2	
KJ174642.1	AIL01014.1	Lasiurus cinereus	USA	VIII	D	2	
KJ174657.1	AIL01029.1	Vulpes vulpes	USA	IX	B	4	
KJ174660.1	AIL01032.1	Nycticeius humeralis	USA	IX	D	3	
KJ174661.1	AIL01033.1	Canis familiaris	USA	VIII	D	3	
KJ174674.1	AIL01046.1	Tadarida brasiliensis	USA	X	C	4	
KJ174676.1	AIL01048.1	Eptesicus fuscus	USA	IX	D	1	
KM492756.1	AIT92127.1	Dog	India	II	D	3	
KJ564280.1	AJF83755.1	Dama dama (fallow deer)	China	V	D	1	
KM594024.1	AKN89648.1	Callithrix jacchus	Brazil	IX	D	1	
KM594025.1	AKN89653.1	Callithrix jacchus	Brazil	IX	C	2	
KM594028.1	AKN89668.1	Eptesicus furinalis	Brazil	IX	D	4	
KM594029.1	AKN89673.1	Eptesicus furinalis	Brazil	IX	D	2	
KM594031.1	AKN89683.1	Myotis nigricans	Brazil	IX	D	2	
KP997032.1	ALC76692.1	Ursus arctos	Russia: Primorsky Krai	VII	B	3	
KT221130.1	ALM97212.1	Dog	China	V	A	1	
KP860204.1	ALW83587.1	Ferret badger	Taiwan	I	A	3	
KP860227.1	ALW83610.1	Ferret badger	Taiwan	I	A	3	
KU739038.1	AMM70620.1	Dog	Burkina Faso	II	D	3	
KU739045.1	AMM70627.1	Bovine	Brazil	X	C	1	
KU888641.1	AMR71127.1	Mephitis mephitis	USA	VI	C	1	
KX148190.1	APD76994.1	Jackal	Iran	VII	C	1	
KX148223.1	APD77159.1	Feline	South Africa	II	B	1	
KX708500.1	ASU55969.1	Dog	Mexico	II	A	2	
MF113393.1	ASV64872.1	Ferret badger	China	V	C	3	
AB563875.1	BAN14114.1	Canis lupus familiaris	Philippines	I	A	1	
AB563915.1	BAN14154.1	Canis lupus familiaris	Philippines	I	B	3	
AB817105.1	BAN51805.1	Homo sapiens	Sri Lanka	I	B	3	
AB817141.1	BAN51841.1	Homo sapiens	Sri Lanka	I	D	1	

The 77 circulating strains we selected and the 6 reference strains were successfully reconstructed as pseudoviruses using the VSV vector. In order to verify their infectivity, the 83 pseudoviruses were quantitated by determining the amount of nucleic acids, and used to infect 293T, Vero and BHK21 cell lines. According to the relative light unit (RLU), the 83 strains were divided into 4 clusters, named A-D (Figure 2(b)), and the infectivity of strains in the four clusters exhibited statistically significant differences (*P* < 0.0001, Figure 2(c)). In all three cell lines, the strains from cluster D had the strongest infectivity, followed by cluster C and B, while cluster A showed the weakest infectivity (Figure 2(c)).

The cluster A group, which had the weakest infectivity, contains 11 strains, 4 of which were isolated from dogs, 6 were isolated from wild animals, while ACR67115.1(Protein Accession Number) were isolated from *white albino mouse* (Table 1). The most infectious cluster D group contained 22 sequences. In addition to 1 reference strains, 13 strains were isolated from wild animals, 6 strains were isolated from domestic animals (feline, ovine and dogs), while AGC12020.1 (Protein Accession Number) and BAN51841.1(Protein Accession Number) were isolated from *Homo sapiens* (Table 1).

### Antigenic evolution of circulating rabies viruses

The infectivity of the 83 pseudoviruses was highest in 293T cells, followed by BHK21 and Vero cells (Figure 2). Accordingly, the pseudovirus neutralization assay was conducted in 293T cells. In our neutralization experiments, the amount of added pseudoviruses was determined based on the TCID50, which was calculated according to the infectivity of pseudoviruses in 293T cells.

**Figure 3.**
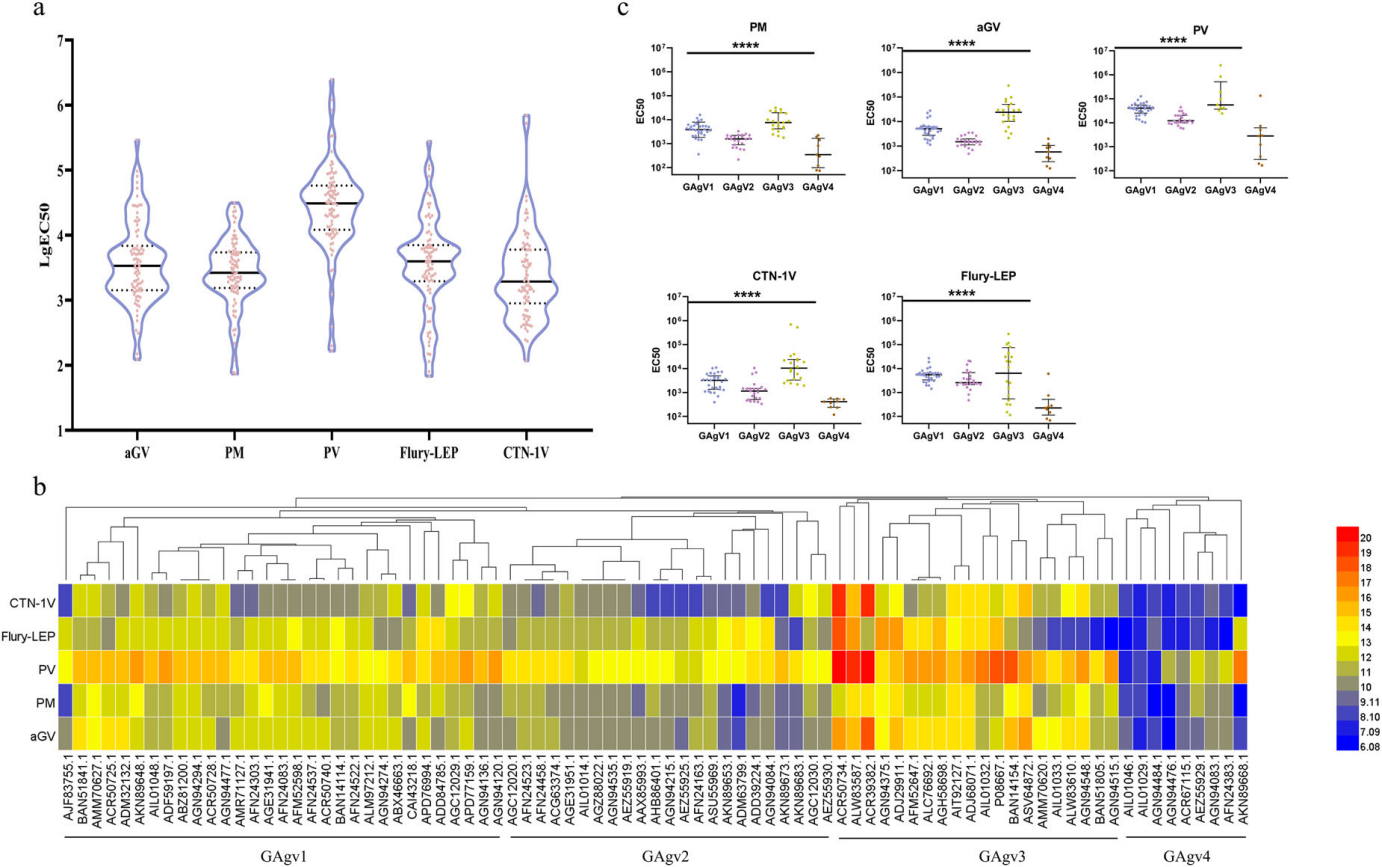
Antigenic evolution of circulating rabies viruses. (a) The neutralization activity of vaccine-elicited sera against rabies pseudoviruses. The EC50 values were determined against a panel of 83 pseudoviruses in our library. The black line represents the median value with interquartile range. (b) Antigenic cluster of 83 representative circulating rabies viruses. (c) The neutralization activity of vaccine-elicited sera against pseudoviruses from different antigenic clusters. The *Y*-axis indicates the EC50 values of different strains and represents the median with the interquartile range. Serum neutralization of different cluster strains was compared using the rank sum test, differences with *P* < 0.05 were considered statistically significant. *P < 0.05; **P < 0.01; ***P < 0.005; ****P < 0.0001.

Then, all 83 pseudotyped viruses were used to determine the neutralizing efficiency of 14 vaccine-elicited sera, including 3, 3, 3, 3, and 2 sera from guinea pigs immunized with vaccines derived from strains PM, PV, Flury-LEP, aGV, and CTN-1V, respectively. Based on the EC50 of the neutralization values, we first calculated the mean EC50 value of the same type of serum against the strains. Although all vaccine-elicited sera could neutralize all 83 pseudotyped viruses (Figure 3(a)), the neutralization levels of the same vaccine-elicited sera against different strains were very different, and the same strains also showed different sensitivity against different sera (Figure 3(a)).

To explore the reason behind the altered sensitivity to the vaccine-elicited sera, we analyzed the antigenic evolution of rabies viruses. We used a hierarchical clustering method to categorize the strains. Figure 3(b) shows the sensitivity to the five types of sera and reveals the advanced features of the evolution of rabies virus antigens. As shown in Figure 3(b), the strains could be divided into four antigenic clusters, named GAgV1-4.

To show the neutralization potency of the vaccine-elicited sera against the strains in the four antigenic clusters, we displayed the detailed neutralization results of each serum against strains from different antigenic clusters. The neutralization results against the different antigenic clusters had significant statistical differences (*P* < 0.0001, Figure 3(c)). All sera had the best neutralizing effect against the GAgV3 strains, followed by GAgV1 and GAgV2, while GAgV4 was the worst (*P* < 0.0001). In addition, as the sera showed significant neutralization differences between the strains in the four antigenic clusters, this proved that the four antigenic clusters we classified were accurate, and the hierarchical clustering method we used in this study was effective (Figure 3(c)).

Thus, the results indicated that the vaccine-elicited sera had the worst neutralization effect on the strains in GAgV4. There were 9 strains in GAgV4, among which AGN94083.1 (Protein Accession Number) and AFN24383.1 (Protein Accession Number) were isolated from skunks in America, AIL01046.1 (Protein Accession Number), AGN94476.1 (Protein Accession Number), AEZ55929.1 (Protein Accession Number) and AKN89668.1 (Protein Accession Number) were isolated from bats in America, AGN944841.1 (Protein Accession Number) was isolated from *Potos flavus* in America, AIL01029.1(Protein Accession Number) was isolated from *vulpes vulpes* in America, while ACR67115.1 was obtained from Asian albino mouse (Table 1). Thus, the strains in GAgV4 were almost all isolated from wild animals in America.

The nine strains in the GAgV4 group almost had stronger infectivity, with ACR67115.1(Protein Accession Number) was at the level of cluster A, Three strains at the level of cluster B, and Four strains at the level of cluster C, while AKN89668.1 (Protein Accession Number) was at the level of cluster D (Table 1).

### The relationship between phylogenetic distance and infectivity or antigenicity

**Figure 4.**
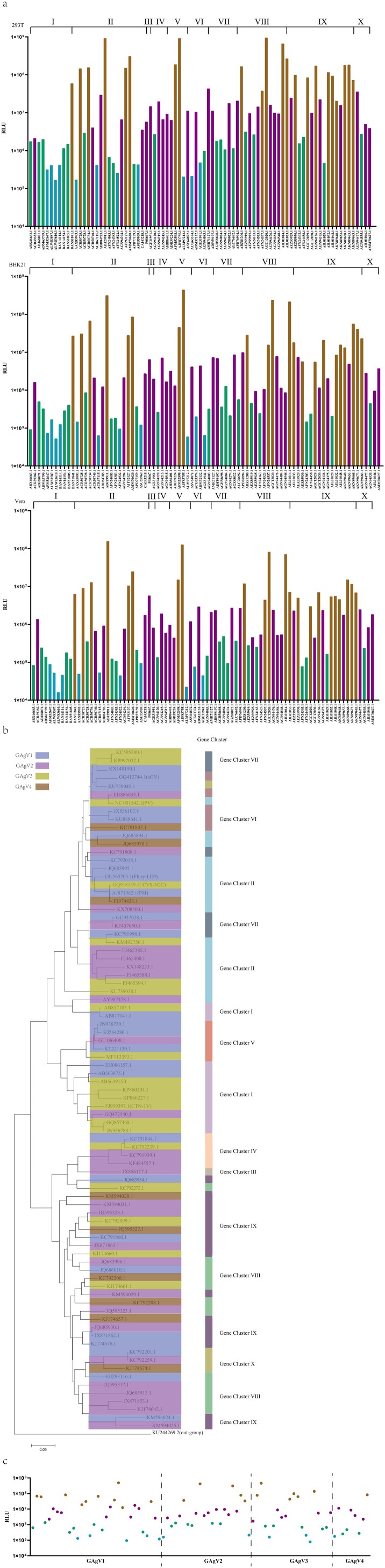
The correlations between phylogenetic relationships and infectivity or antigenicity. (a) The relationship between phylogenetic clusters and infectivity clusters. Assays in 293T, BKH21 and Vero cells reflect the relationship between phylogeny and infectivity. The X-axis shows the gene accession number of each strain, the Y-axis shows the RLU values (mean ± SD), the differently coloured columns show the different infectivity clusters. The colour scheme was the same as in Figure 2(c). (b) The correlation between phylogenetic relationships and antigenicity. A phylogenetic tree of the viral strains was constructed based on sequence similarity. The information on antigenic, phylogenetic, and geographic clusters is showed in the phylogenetic tree. (c) The relationship between infectivity and antigenicity. The X-axis is split in four parts according to antigenic clusters, the Y-axis shows the median of the neutralization EC50 value of 14 vaccine-elicited sera, and the differently coloured spots indicate the different infectivity clusters. The colour classification is the same as in figure 2c.

We tried to find the connection between phylogenetic relationships and viral infectivity. Unfortunately, there was no obvious connection between phylogenetic relationships and virus infectivity. Most strains in the ten gene clusters belonged to the infectivity cluster C, but some strains that were randomly distributed among the ten gene clusters belonged to the infectivity clusters A, B or D. For example, 11 strains in infectivity cluster A were located in gene clusters I, II, V and VI, (Figure 4(a)). This suggests that the phylogeny had no direct relationship with infectivity.

Next, we investigated the antigenic basis of the genetic cluster structure, and unexpectedly found that the antigenicity of the strains in each gene cluster was not unique. Although most strains in the ten gene clusters belonged to the antigenic cluster GAgV1, a few strains that were randomly distributed among the ten gene clusters belonged to the antigenic clusters GAgV2-4 (Figure 4(b)). For example, nine strains in GAgV4 were located in gene clusters II, VIII, IX, and X (Figure 4(b)). This means that the phylogeny also has no direct relationship with antigenicity.

When the relationship between the clusters of infectivity and antigenicity was analyzed, there was also no obvious correspondence. Most strains in the antigenic cluster GAgV2 belong to infectious cluster C, while a few strains belonged to infectivity clusters A, B and D (Figure 4(c)). The antigenic cluster GAgV4 that had the least strains also belongs to infectivity clusters A-D (Figure 4(c)).

### Specific mutations affecting infectivity and antigenicity

**Figure 5.**
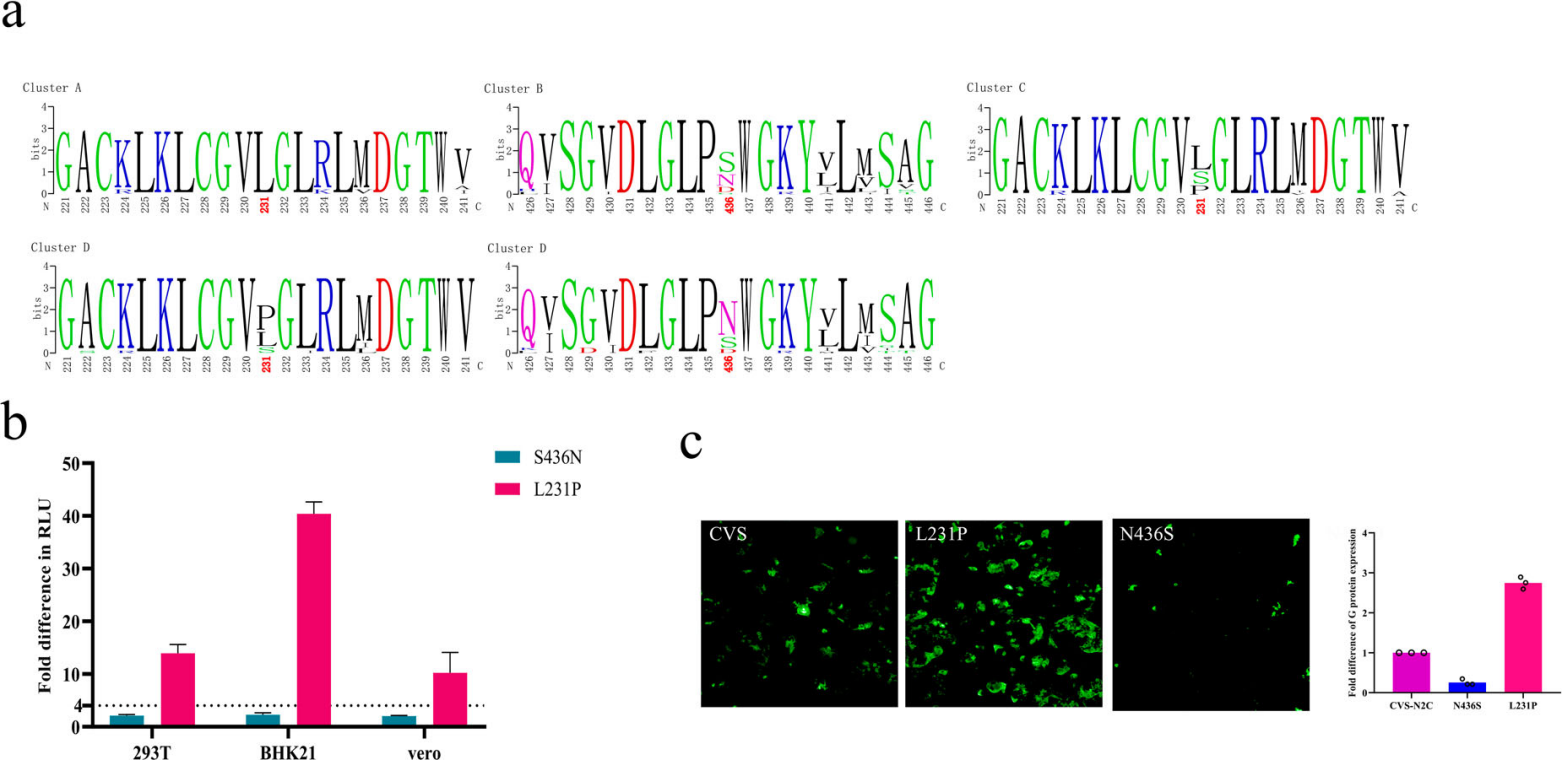
Specific mutations affecting infectivity. (a) Cluster-specific amino acid substitutions. The sequences were aligned according to the four infectivity clusters and the significant amino acid mutations are marked in red. (b) The infectivity of four mutants in 293T, BHK21, and Vero cells. RLU values of the mutants were compared with the reference strain CVS-N2C. A 4-fold or greater difference was considered significant; all data are the means ± SEM from at least three replicates. Figure c. the G protein expression of three mutants in Vero cells. The y axis represents the G protein expression fold changes of the mutants when compared with the reference strain CVS-N2C.

We divided the 83 strains into four infectivity clusters based on the RLU values. When the amino acid sequences of strains belonging to different infectivity clusters were aligned and analyzed, we found that there were significant single amino acid point mutations that could distinguish between infectivity cluster D and the other three infectivity clusters. The difference between the infectivity clusters A and D was mainly located at residue 231 (L→P; Figure 5(a)). The main mutations distinguishing between the infectivity clusters B and D was occurred at residues 436 (Figure 5(a)). Residue 436 was mainly S in cluster B, but it changed to N in most strains from cluster D (Figure 5(a)). The difference between the infectivity clusters C and D was mainly located at the epitope I at residue 231 (Figure 5(a)). Residue 231 was L in cluster C, but it changed to P in most strains from cluster D (Figure 5(a)).

We incorporated these 2 amino acid mutations that can affect infectivity into pseudoviruses using CVS-N2C as backbone for high throughput pseudovirus assays. We then tested the infectivity of the mutant pseudoviruses in 293T, BKH21 and Vero cells. More than 4-fold differences in RLU compared with the reference strain CVS-N2C were deemed significant. Compared with CVS-N2C, the infectivity of the L231P mutant was enhanced by 14, 40 and 10 times in 293T, BHK21 and Vero cells, respectively. The infectivity of S436N was slightly increased within four times (Figure 5(b)). Furthermore, we used plasmids to transfect Vero cells and detected G protein expression by immunofluorescence (Figure 5(c)). It was observed that after plasmid transfection, the expression of G protein was significantly increased in the cells transfected with the L231P mutant, which may be the reason for the enhanced infectivity of the L231P pseudovirus (Figure 5(c)). It was also worth noting that the pseudovirus of N436S reduced the expression of G protein, but the mutation we found that can increase infectivity was S436N. The reason for the enhanced infectivity of S436N pseudovirus was, therefore, also the increased expression of G protein (Figure 5(c)).

**Figure 6.**
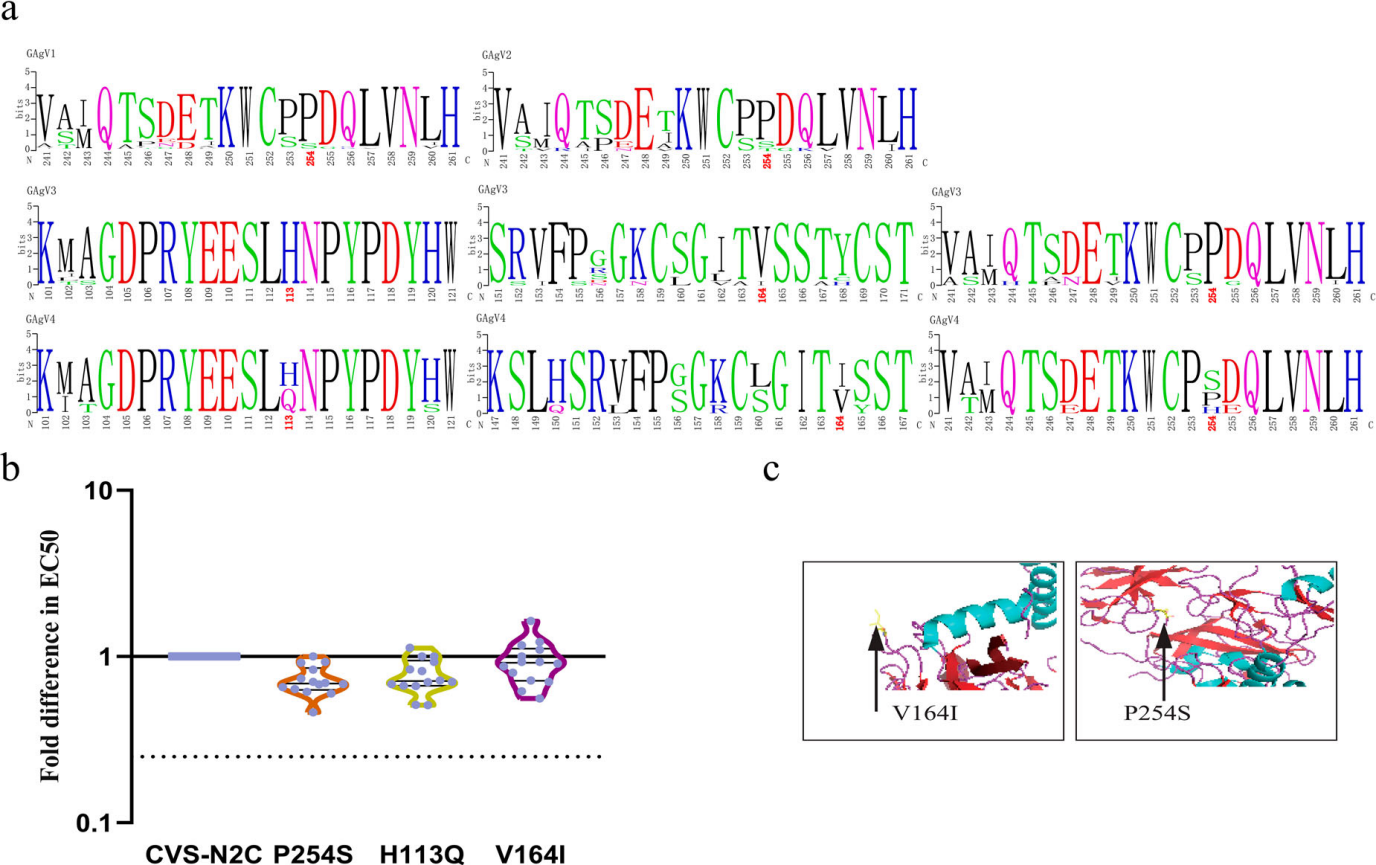
Specific mutations affecting antigenicity. (a) Cluster-specific amino acid substitutions. The sequences were aligned according to the four antigenic clusters and the significant amino acid mutations are marked in red. (b) Decreasing reactivity of pseudotyped viruses carrying the four mutations to the 14 vaccine-elicited sera. The ratio of EC50 between mutants and the reference strain (CVS-N2C) was calculated and 4-fold or greater differences were considered significant. The column height represents the median of the EC50; error bars represent the SEM. (c) Structural simulation. The yellow position and the arrow show the amino acid mutation.

We next aligned and analyzed the amino acid sequences of strains belonging to different antigenic clusters (Figure 6(a)) and found that there were significant single amino acid point mutations between GAgV4 and the other three antigenic clusters. The main mutations that could distinguish between GAgV1 and GAgV4 occurred at residue 254. This residue was mainly P in GAgV1, but it changed to S in GAgV4 (Figure 6(a)). The main difference between the GAgV2 and GAgV4 groups was also mainly at residue 254 (P→S; Figure 6(a)). In addition, the differences between GAgV3 and GAgV4 were mainly at residues 113, 164, and 254 (Figure 6(a)). Residue 113 was H in GAgV3, but it changed to Q/H in most strains from GAgV4. Residue 164 was V in GAgV3 and changed to I/V in most strains from GAgV4, while residue 254th changed from P to S (Figure 6(a)). Notably, residue 254 distinguished a single cluster from all aligned antigenic clusters (Figure 6(a)).

We incorporated the three amino acid mutations that can affect serum neutralization sensitivity into pseudoviruses using CVS-N2C as backbone for high throughput neutralization assays. We determined the sensitivity of the strains with the three amino acid changes to 14 vaccine-elicited sera. More than 4-fold differences in EC50 compared with the reference strain CVS-N2C were deemed significant. The H113Q and P254S mutants both demonstrated slightly altered sensitivity to all 14 vaccine-elicited sera, when compared with the reference strain CVS-N2C, while the V164I mutant exhibited slightly altered reactivity to 9 of 14 vaccine-elicited sera (Figure 6(b)).

## Discussion

As an RNA virus, the rabies virus is highly prone to developing mutations during the normal replication process. Closely monitoring the antigenic evolution of circulating virus strains is of unquestionable importance for the development of vaccines and therapeutics. Here, we conducted an epidemiological and phylogenetic analysis of 2896 rabies G gene sequences and found that the viruses evolution showed phylogenetic patterns reflecting both geography and host species. This is consistent with a previous study [37]. Although the research conducted by Carnieli et al. was limited to Northeast Brazil [31], the evolution of rabies viruses in this region still has geographic and species specificity. When the scope was expanded to Brazil, it was found that rabies virus evolution was mainly driven by adaptation to the host species [21]. At the same time, evolutionary research in Europe also indicated that rabies virus evolution had geographical specificity [26].

In this study, we found evidence of cross-species transmission between ferret-badger and dog rabies viruses. This is notable, since cross-species transmission of rabies virus usually only leads to sporadic cases and does not spread further [21, 25, 38, 39]. For example, human rabies has symptoms, usually leading to fatal results, but no subsequent transmission. However, occasionally rabies virus can establish a productive infection in a new host species [40–42] and cause rabies virus epidemics [26, 43]. When rabies virus spreads across species, it is inevitable that adaptive mutations will be produced during evolution. Over time, genetic mutations gradually accumulate, and this accumulation will eventually affect the protective effect of vaccines.

When analyzing infectivity, we divided the virus strains into 4 clusters according to their infectivity in 293 T, BHK21 and Vero cells, among which cluster D had the most robust infectivity. By analyzing the amino acid sequences of the virus strains, we found two mutations that significantly enhance the infectivity of the strain, including S436N and L231P. However, these mutations did not alter the neutralizing reactivity of the vaccine-elicited serum and HRIG over 4 times [44]. Therefore, these amino acid sites provide the possibility for the construction of high titre pseudovirus, especially the outside antigenic sites S436N.

To explore the antigenic evolution of rabies virus, we successfully constructed a rabies antigen evolution model, and the strains were finally divided into four antigenic clusters. Most vaccine-elicited sera had poor neutralization activity against the strains from GAgV4. These strains were almost all isolated from American wild animals, especially bats and skunks. Although in previous studies, antigenic characterization was mainly based on monoclonal antibodies against the viral N protein, many antigenic variants of bats and skunks were also found. The antigenic variants of bat mainly belonged to NAgV3, NAgV4, NAgV9, NAgV11 and ND while those of skunk belonged to NAgV8 and NAgV10 [22, 32, 33, 45].

Bats and skunks are both unique hosts of the American rabies virus, and both play an important role in prevalent cycles of viral endemism [14, 15, 37, 46, 47]. In Mexico, Brazil and Ecuador, there were reports of human rabies deaths that were caused by a variant strain of the bat rabies virus [48, 49]. In addition to humans, bat rabies can also cause spillover infection in domestic animals such as cows [25, 48, 50]. Thus, we should strengthen the monitoring of the transmission dynamics and epidemic trend of wild animal RABVs in America, update the virus sequence library in time, regularly study the antigenic evolution of wild animal RABVs, and find out those mutations that may affect the neutralization activity of vaccine-immune sera.

We aligned the sequences in the four antigenic clusters, and found that there were significant amino acid differences between GAgV4 and the other three antigenic clusters, encompassing the mutations H113Q, V164I, and P254S. We used a structural model of the G protein [51] to simulate some amino acid mutations. Among these, the V164I and P254S mutations occurred in the loop of the G protein (Figure 6(c)). At position 254, the non-polar hydrophobic amino acid Pro transitioned into the neutral amino acid Ser or the alkaline amino acid His, causing the net charge of the amino acid to change, affecting G protein function. The same is true for H113Q, which changes a positively charged into a polar amino acid.

In this study, there was no obvious connection between the phylogeny and infectivity or antigenicity of rabies virus. This evolutionary pattern is significantly different from other infectious viruses, such as H3N2 and H1N1 [52, 53]. We also found that single-point amino acid mutations promote changes of antigenicity and infectivity. It is possible that single-point amino acid mutations are not as important for phylogenetic relationships as multi-point mutations, and can be easily neglected in conventional evolutionary analysis in spite of their pivotal role in the infectivity and antigenic evolution of rabies, resulting in inconsistencies between phylogeny and antigenicity or infectivity.

Taken together, we comprehensively analyzed the phylogenetic relationships, infectivity and antigenicity of rabies virus isolates from around the world. No close relationship among them was found. However, specific mutations that affect infectivity or antigenicity were found. This indicates that the analysis of phylogenetic relationships cannot predict the infectivity and antigenicity of rabies viruses. Thus, the infectivity and antigenicity should be specifically monitored in addition to phylogenetic relationships in the future. This study lays a foundation for further development and updating of rabies virus vaccines.

## Materials and methods

### Cell culture

The 293T (CRL-3216), Vero (ATCC, CCL-81) and BHK21 cells (ATCC, CCL-10) were obtained from the American Type Culture Collection. All cell lines were cultured in Dulbecco’s modified Eagle’s medium (HyClone, Logan, UT) with 100 U/mL of penicillin–streptomycin solution (GIBCO, Grand Island, NY), 20 mM N-2-hydroxyethylpiperazine-N-2-ethane sulphonic acid (GIBCO), and 10% fetal bovine serum (PAN-Biotech, Aidenbach, Germany) at 37°C in a humidified atmosphere comprising 5% CO_2_.

### Construction, preparation, and titration of RABV pseudoviruses

The RABV G protein sequences in Table 1 were downloaded from NCBI, synthesized and cloned into the backbone plasmid pcDNA3.1(+) by General Biological Systems (Anhui, China). All plasmids were introduced into Trans5α Chemically Competent Cells (TransGen Biotech, Beijing, China) for preservation and amplification, and extracted using the Plasmid Plus Midi Kit (Qiagen, Dusseldorf, Germany). The preparation and titration methods for rabies pseudovirus were described previously [54]. In the VSV pseudovirus system, 293T cells were transfected with all RABV glycoprotein-expressing plasmids using Lipofectamine 3000 (Invitrogen, Carlsbad, CA), according to the manufacturer’s instructions. After 24 h, the transfected cells were infected with G *ΔG-vesicular stomatitis virus (Kerafast, Boston, MA). After 1 h, the 293T cells were washed with PBS (GIBCO) three times and then new complete culture medium was added. After 24 h, the pseudoviruses in the culture supernatant were harvested, filtered using a 0.45 µm pore-size membrane (Millipore, Boston, MA) and stored at −80°C. For the rabies pseudovirus titration, a 5-fold initial dilution was carried out of 96-well culture plates, followed by serial 5-fold dilutions, with the last column serving as the cell control and containing only cells without pseudovirus. Then, a predetermined concentration of 293T cells were added into the 96-well plates. And the surrounding wells contained PBS to prevent the liquid from evaporating. The plates were incubated in a 5% CO_2_ incubator at 37°C. After 24 h, the luminescence values (PerkinElmer, Waltham, MA) were measured, and the TCID50 was calculated as described previously [55].

### Quantification of pseudotyped virus particles by RT–PCR

All the pseudotyped viruses were purified through ultracentrifugation at 100,000× *g* for 3 h, and 140 µl of the purified pseudotyped virus suspension was used to extract viral RNA using the QIAamp Viral RNA Mini Kit (Qiagen, Dusseldorf, Germany). This RNA was used as template for reverse transcription to obtain cDNA using the SuperScript III First-Strand Synthesis System for RT–PCR kit reagent (Invitrogen, Carlsbad, CA). Virus quantification through real-time PCR was performed using the TB Green Premix Ex Taq II kit (TaKaRa, Kyoto, Japan), following the manufacturer’s instructions. The P protein gene of VSV virus was cloned into the vector pCDNA3.1(+) as a plasmid standard, and the viral copy number was calculated accordingly.

### RABV pseudotyped virus infection assay

Based on the quantitative RT–PCR results, we normalized the pseudotyped virus particle suspensions to the same concentration. After normalization, 100 μL of the pseudotyped virus with 15-fold dilution was added to the wells of a 96-well cell culture plate, after which trypsin-digested cells (3 × 10^4^/100 μL) were added into each well. The plates were then incubated at 37°C with 5% CO_2_. After incubation for 24 h, chemiluminescence detection was performed as described in the titration of pseudotyped viruses. Each group contained at least three independent replicates.

### *In vitro* pseudovirus-based neutralization

Neutralization was measured by the reduction in RLU values similar to Rift valley fever pseudovirus, as described previously [55]. Briefly, RABV pseudovirus was incubated with serial dilutions of vaccine-elicited sera (62.5-fold in the initial dilution, then 5-fold serially diluted) for 1 h at 37°C in an incubator with 5% CO_2_. After 1 h, 100 µL of cells was added to each well. Following co-incubation for 24 h at 37°C with 5% CO_2_, the luminescence values were measured using a luminometer (PerkinElmer, Waltham, MA), and the reduction values were calculated by comparison with the control wells after 24 h. The EC50 of sera were calculated with the Reed–Muench method.

### Vaccine-elicited sera

Animals were handled under institutional guidelines for laboratory animal care and use of NIFDC (Beijing, China), and the Animal Care and Use Committee at the NIFDC approved the study protocol.

The vaccine DNA or pseudovirus including PV (GenBank accession no: P08667.1), PM (GenBank accession no: CAI43218.1), Flury-LEP (GenBank accession no: ADD84785.1), aGV (GenBank accession no: ADM32132.1), and CTN-1V (GenBank accession no: ACR39382.1) were used to immunize guinea pigs. On week 0, female guinea pigs (*n* = 3/group) were immunized intramuscularly with vaccine-DNA (50 µg of each guinea pig). On week 2, the female guinea pigs were abdominally injected with pseudovirus (2000 pg of each guinea pig) according to the DNA type. On week 4, blood samples were harvested. Serum samples were stored at −80°C, thawed and heat-inactivated at 56°C for 0.5 h before used.

### Construction, titration, infectivity and neutralization of pseudoviruses carrying the identified amino acid mutations

The CVS-N2C glycoprotein expressing plasmid psCMV.CVS-N2C was constructed as described previously [44] and used as template for site-directed mutagenesis. The pseudovirus preparation, titration, infectivity and neutralization assays were the same as described for the wild-type pseudoviruses.

### Determination of G protein expression–high content cell imaging

The Vero cell concentration was adjusted to 1 × 10^5^ cells/mL in 96-well plates (PerkinElmer, Waltham, MA) and incubated for 12 h in a 5% CO_2_ incubator at 37°C. After 12 h, the Vero cells were transfected with 0.4 µg of plasmid DNA using Lipofectamine 3000 (Invitrogen, Carlsbad, CA), according to the manufacturer’s instructions, and incubated for 30–36 h. Then, the medium was removed, and the cells were fixed with 4% paraformaldehyde at room temperature for 15 min, washed twice with PBS (GIBCO), permeabilized with 0.25%TritonX-100 at room temperature for 10 min, washed twice with PBS, and blocked with 1% BAS-containing PBS solution for 30 min at room temperature. Then, 06-2A12 antibody with a final concentration of 1 mg/L was used as the primary antibody, and incubated at room temperature for 1–2 h. Then, the cells were washed 3 times with PBS solution containing 1% BAS (sigma, Saint Louis, MO), followed by incubation at room temperature for 1 h with FITC-labeled goat anti-human IgG (abcam, Cambridge, UK) diluted 500 times with 1% BSA as secondary antibody. Then, the cells were washed with PBS, and nuclei were counter-stained at room temperature for 15 min in the dark with DAPI (Beyotime Biotech, Beijing, China) diluted 1000 times in PBS, Operetta CLS (PerkinElmer, Waltham, MA) was used to record high-content photographs, and Harmony 4.9 software was used to process the images.

### Phylogenetic tree analysis

All 2897 gene sequences (2896 rabies sequences and 1 out-group sequence) were downloaded from NCBI. Multiple sequence alignments of all sequences were performed using MUSCLE, after which a maximum-likelihood tree was constructed using RAxML. Because of the large amount of calculation, the MPI version was used for distributed calculation, the PROTGAMMAAUTO model was used for construction, and the number of bootstrap iterations was set to 1000. For the 84 sequences (83 rabies sequences and 1 out-group sequence) used in the serum neutralization experiments, multiple sequence alignments were displayed in MEGA. The phylogenetic tree was further modified using the online software iTOL (https://itol.embl.de/tree/). The smaller phylogenetic tree with 84 sequences was also constructed using this method.

### Processing of serum neutralization data

GraphPad Prism 8.0 was used to analyze the neutralization activity of all the sera against the 83 strains. When dimensionality reduction analysis was performed on the serum neutralization data of the 83 strains, we used the hierarchical clustering method to cluster the strains. The clustering results were displayed using HEML software.

### G protein amino acid substitutions and structure simulation

BioEdit software was used to find amino acids that differed between different antigen clusters. The G protein structure simulation was done using PyMol software (PyMOL Molecular Graphics System, Version 2.2.0, Schrödinger, LLC.).

### Statistical analysis

In the vaccine-elicited sera neutralization assay, statistical significance was determined using SPSS 20.0. Differences in the sera potency tests against the strains in the four antigenic clusters were analyzed using the rank sum test, * *P* < 0.05, ** *P* < 0.01, *** *P* < 0.005, **** *P* < 0.001.

## Supplementary Material

Supplemental Material
