# Supplementary figures and images for: Analysis of the evolution, infectivity and antigenicity of circulating rabies virus strains
Source: Emerg Microbes Infect. 2022 Jun 1;11(1):1474–87. doi: 10.1080/22221751.2022.2078742 (PMC9176641; doi:10.1080/22221751.2022.2078742)

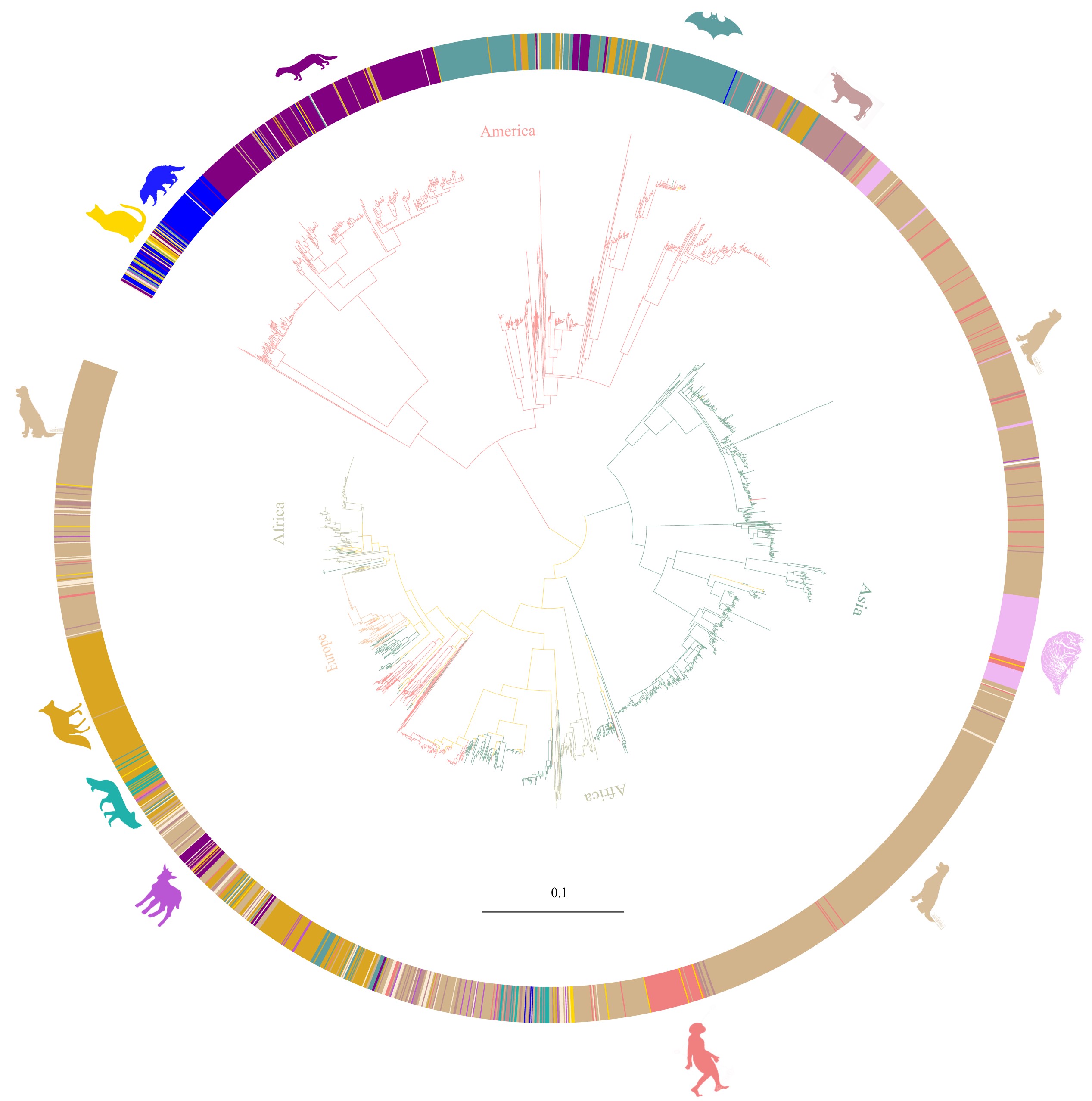

Supplement: Supplemental Material [file TEMI_A_2078742_SM5068.jpg]
